# Applications of Lgr5-Positive Cochlear Progenitors (LCPs) to the Study of Hair Cell Differentiation

**DOI:** 10.3389/fcell.2019.00014

**Published:** 2019-02-19

**Authors:** Danielle R. Lenz, Niliksha Gunewardene, Dunia E. Abdul-Aziz, Quan Wang, Tyler M. Gibson, Albert S. B. Edge

**Affiliations:** ^1^Department of Otolaryngology, Harvard Medical School, Boston, MA, United States; ^2^Eaton-Peabody Laboratories, Massachusetts Eye and Ear, Boston, MA, United States; ^3^Harvard Stem Cell Institute, Cambridge, MA, United States

**Keywords:** Lgr5, differentiation, proliferation, hair cells, supporting cells, cochlea, epigenetics

## Abstract

The mouse cochlea contains approximately 15,000 hair cells. Its dimensions and location, and the small number of hair cells, make mechanistic, developmental and cellular replacement studies difficult. We recently published a protocol to expand and differentiate murine neonatal cochlear progenitor cells into 3D organoids that recapitulate developmental pathways and can generate large numbers of hair cells with intact stereociliary bundles, molecular markers of the native cells and mechanotransduction channel activity, as indicated by FM1-43 uptake. Here, we elaborate on the method and application of these Lgr5-positive cochlear progenitors, termed LCPs, to the study of inner ear development and differentiation. We demonstrate the use of these cells for testing several drug candidates, gene silencing and overexpression, as well as genomic modification using CRISPR/Cas9. We thus establish LCPs as a valuable *in vitro* tool for the analysis of progenitor cell manipulation and hair cell differentiation.

## Introduction

Hearing loss is the most prevalent form of sensory loss, affecting 466 million people worldwide (World Health Organization^1^). Deafness can be caused by genetic and environmental factors, mostly affecting the non-regenerating hair cells of the inner ear, which are responsible for translation of sound into a neural signal. In recent years many attempts have been made to generate an *in vitro* model that adequately represents native hair cells, to enable molecular analysis of their differentiation and maturation. These attempts included organoid generation from both human and mouse embryonic stem cells (Oshima et al., 2010; Koehler et al., 2013; Ronaghi et al., 2014; Costa et al., 2015; Ding et al., 2016) induced pluripotent stem cells (Oshima et al., 2010; Koehler et al., 2017) and reprogrammed otic progenitors and supporting cells (Kwan et al., 2015; Roccio et al., 2015; Walters et al., 2015). However, despite considerable success, a low yield of mostly immature hair cells has been obtained in these systems.

During embryogenesis, the Notch and Wnt signaling pathways play an essential role in the development of the sensory epithelium. Moreover, activation of the Wnt pathway and inhibition of the Notch pathway have been demonstrated to induce partial regeneration of hair cells (Mizutari et al., 2013; Shi et al., 2014). Lgr5 is a cell membrane receptor of the Wnt-pathway, which has come to be recognized as a stem-cell marker in the inner ear. Supporting cells expressing Lgr5 transdifferentiated into hair cells postnatally under specific conditions (Groves, 2010; Chai et al., 2012; Shi et al., 2012; Bramhall et al., 2014). Our lab recently established a protocol for expansion of Lgr5-positive cochlear cells as organoids, to obtain Lgr5-positive cochlear progenitors (LCPs) in large numbers *in vitro*, using a combination of growth factors and small molecules. LCPs could then be efficiently differentiated into hair cells (McLean et al., 2017). In the inner ear sensory epithelium, hair cells and supporting cells develop from a common sensory progenitor. Similar to other *in vitro* epithelial-derived organoid models, such as the intestine, this model is based on progenitor cells that retain their lineage of origin and thus serves as a model of development. LCPs are generated by enriching and expanding the Lgr5-positive cell population, establishing a semi-pure progenitor culture. Differentiation of LCPs was observed after combined treatment with a Notch-inhibitor and a Wnt-activator, supporting their potential as a model for *in vivo* differentiation. The Lgr5-positive fraction of the organoids differentiated into a population expressing hair cell markers, including *Atoh1*, *Myo7a*, and *Tmc1*, and possessing apical stereociliary elongation and FM1-43 uptake, indicating functional transduction channels (McLean et al., 2017).

Recent studies in cellular reprogramming have revealed an important role for epigenetic modifications in influencing stem-cell pluripotency and differentiation (Krishnakumar and Blelloch, 2013). Epigenetic modifiers of histones and DNA methylation alter chromatin structure and DNA accessibility, thus influencing gene expression and cell fate. The current trend in epigenetic research is progressively expanding to include both evaluation of the epigenetic cell state during development and later perturbations for regenerative purposes (He et al., 2018). In the inner ear, epigenetic regulation of development and regeneration has only recently been studied, limited by the low number of cells in the cochlear sensory epithelium, relative to the number of cells required for assays of epigenetic marks (Stojanova et al., 2015; Abdolazimi et al., 2016). As such, accumulation of hair cell and supporting cell samples from multiple animals has been necessary for the execution of a single experiment, thus increasing the variability between experiments and restricting their use for downstream translational studies. Due to the complexities of *in vivo* analysis, an *in vitro* model is needed for initial evaluation of epigenetic changes, leading to a complete analysis at the histone and gene levels. Additionally, it has recently become possible to directly perturb epigenetic marks at specific genomic loci by genetically fusing epigenetic effector proteins to programmable, sequence-specific DNA binding proteins such as the RNA-guided nuclease CRISPR/Cas9. Epigenetic modifications that have been accomplished with these tools include targeted DNA methylation (Rivenbark et al., 2012), histone deacetylation and demethylation (Kearns et al., 2014), and histone acetylation (Hilton et al., 2015). Due to the scalability of RNA synthesis, it is also possible to perform high-throughput screening of several genomic elements (Gilbert et al., 2014) given a sufficient number of cells. Execution of such experiments requires a robust and reliable *in vitro* model, as recently demonstrated using organoid models (Driehuis and Clevers, 2017).

A major advantage of the LCP system is the ability to generate organoids from various genetic mouse models, thus enabling genetic-manipulation using Cre/loxP, tet-on and tet-off systems as well as lineage tracing. Nevertheless, there is still an ongoing need to examine and manipulate gene expression in the absence of a mouse model. Here, we demonstrate the use of LCPs as a tool for efficient testing of epigenetic and other candidate drugs to assay their effect on both proliferation and differentiation as a mean of exploring their role in sensory epithelia development and maturation. In addition, we describe a lentiviral transduction protocol that enables introduction of foreign DNA for knockdown, overexpression or CRISPR/Cas9-mediated genome editing, demonstrating the potential of LCPs for the study of cell signaling, development and regeneration.

## Materials and Methods

### Mice

All animal experiments were conducted according to National Institute of Health guidelines and were approved by the Massachusetts Eye and Ear Institutional Animal Care and Use Committee. LCPs were generated from *Lgr5-EGFP-IRES-CreER* mice (The Jackson Laboratory, strain 008875) (Barker et al., 2007) for proliferation analysis; from *Atoh1-nGFP* mice (provided by Dr. Jane Johnson) (Lumpkin et al., 2003) for differentiation analysis and from *Sox2-CreER* mice (provided by Konrad Hochedlinger) (Arnold et al., 2011) crossed to *CAG-flox-tdTomato* mice (The Jackson Laboratory, strain 007909) and *CAG-flox-Cas9* mice (The Jackson Laboratory, strain 026816) for lentiviral induced CRISPR/Cas9 mediated silencing.

### Cochlear Dissection

**FIGURE 1:**
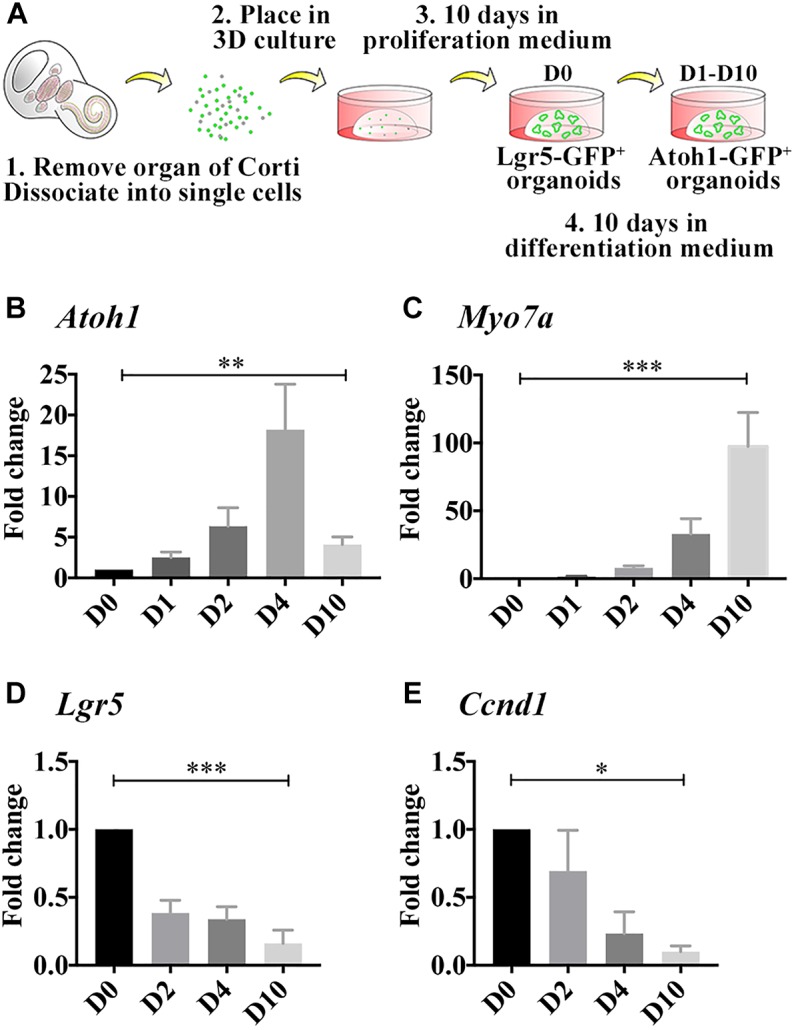
Differentiated LCPs resemble native hair cells. **(A)** Schematic representation of LCP generation and 3D culture in Matrigel. Sensory epithelial cells are harvested from newborn cochleae, dissociated and plated in 3D Matrigel droplets. When harvested from *Lgr5-EGFP-IRES-CreER mice*, Lgr5-positive organoids are fluorescent during the expansion phase, while when harvested from *Atoh1-nGFP* mice, Atoh1-positive organoids are fluorescent during differentiation (modified from McLean et al., 2017). **(B–E)** Expression analysis of whole-culture samples, using qPCR demonstrates *Atoh1* **(B)** and *Myo7a* **(C)** expression patterns that resemble *in vivo* regulation during development. *Lgr5* **(D)** and *Ccnd1* **(E)** expression is reduced as expected from differentiated cells that are exiting the cell cycle. Results are presented as the average fold change ± SEM, between D0, which reflects the first day on which differentiation medium is applied, and days 1–10 (D1–D10); ^∗^*p*-value ≤ 0.05; ^∗∗^*p*-value ≤ 0.01; ^∗∗∗^*p*-value ≤ 0.005 calculated using one-way ANOVA for D0–D10.

For optimal dissection yield and maximal LCP proliferative capacity, cochleae from at least three P2-P4 mouse inner ears were dissected in phenol red-free HBSS with calcium and magnesium (Gibco, #14025134), the cartilage was opened, the stria vascularis was removed and the sensory epithelia detached and incubated in a 100–200 μl droplet of Matrisperse Cell Recovery Solution (Corning) for 1 h at room temperature. This non-enzymatic solution disintegrates the extracellular matrix of the sensory epithelia, enabling the subsequent separation of the hair cells and supporting cells from the mesenchyme and neurons, using a stripping method, by which one forceps is holding the sensory epithelium, while the other is gently removing the layer of hair cells and supporting cells. The forceps holding the intact sensory epithelium remained with the mesenchyme and neurons that were removed from the dish, while the hair cells and supporting cells remained in the dish for subsequent collection. The cells were then collected, centrifuged 5 min at 0.5 ×*g* and incubated in TrypLE (Gibco) for 20 min at 37°C. After additional centrifugation for 5 min at 0.5 ×*g*, the dissociated cells were suspended in HBSS, triturated 50–80 times using a 200 μl pipette tip and strained using a 40-micron cell strainer to produce a single-cell suspension. The cells were then centrifuged again for 5 min at 0.5 × *g* and the pellet was resuspended in 100% Matrigel Basement Membrane Matrix (Growth factor reduced, LDEV-free, Corning). Droplets (30–40 μl) of Matrigel were placed in the center of each well of a 24-well plate, 1 droplet per well (Figure 1A). For optimal confluence, the number of droplets was equal to the number of originally dissected cochleae. For rapid Matrigel polymerization, the plate was incubated for 5–10 min at 37°C, after which the droplets were covered in medium. Evaluation of sphere growth and confluence was conducted with bright field imaging using a fluorescence dissecting scope (Zeiss).

### Expansion and Differentiation of LCPs

To induce proliferation of supporting cells and generation of spheroid colonies, a basal DMEM/F12 medium, containing N2/B27 serum free supplements, fungizone, ampicillin (50 μg/ml) and HEPES was supplemented with bFGF, EGF, and IGF (50 ng/ml each); the antioxidant 2-phospho-L-ascorbic acid (pVc, 280 μM); the HDAC inhibitor valproic acid (VPA, 1 mM) and the Gsk-3β inhibitor CHIR99021 (CHIR, 3 μM). The medium was changed every other day for 10 days. To induce differentiation, the expansion medium was replaced by basal DMEM/F12 medium supplemented with the Notch inhibitor LY411575 (LY, 10 μM) and CHIR (3 μM). The first day of applying differentiation medium is referred to as D0. The medium was changed every other day for the duration of the experiment.

### Design and Generation of Viral Vectors

A fluorescent marker was introduced into a plasmids containing the sgRNA lentiviral transfer vector by digesting pLV hU6-sgRNA hUbC-dCas9-KRAB-T2a-Puro (Thakore et al., 2015) (Addgene #71236) at the PacI restriction sites and inserting a gene block (IDT) containing an optimized chimeric gRNA scaffold (Chen et al., 2013). The resultant plasmid was digested with XbaI and EcoRI. eGFP or tdTomato were ligated into these sites to generate pLV U6-sgRNA UbC-GFP (Addgene #106948) or pLV U6-sgRNA UbC-tdTomato (Addgene #106949), respectively.

sgRNAs targeting *Notch1* were synthesized as primer pairs (Integrated DNA Technologies), treated with polynucleotide kinase (NEB), and annealed by cooling from 95 to 25°C at a rate of 0.1°C/s. Annealed primer pairs were ligated into pLV U6-sgRNA UbC-GFP digested with Esp3I (Thermo). All plasmids, including the sgRNA plasmids targeting *Notch1* (Addgene #106950 – 106952) have been made available on the Addgene repository. Three protospacer sequences were designed: Notch1-1 – CTACCTCTTGCGGCGAGCGC; Notch1-2 – GTGTGTGAGTACCGCCCCTG; Notch1-3 – CCAAGTGGGACCTGCCTGAA. Targets that did not start with a guanine were supplemented with an additional “G” at the 5^′^ end of the protospacer to enable expression from the U6 promoter.

The lentivirus was generated as described previously (Barde et al., 2010) using a third-generation plasmid system. For this purpose, HEK293T cells were maintained in DMEM with 10% fetal bovine serum (FBS, Biological Industries). HEK293T cells were seeded to approximately 80% confluency in 15-cm dishes. The packaging plasmids pRSV-Rev (Addgene #12253) and pMDLg/pRRE (Addgene #12251), the VSV.G envelope plasmid pMD2.G (Addgene #12259), and the third-generation transfer plasmid were transfected into the HEK293T cells using calcium phosphate, with 3.65, 10.95, 7.9, and 22.5 μg, respectively, per 15-cm dish transfected. Transfection was performed late in the afternoon. Early the next morning, the cell culture medium was changed to fresh DMEM with 10% FBS. Virus-containing supernatant was harvested 8 h later and again the next morning and pooled. Collected supernatant was passed through a 0.22-micron filter and concentrated by ultracentrifugation at 100,000 ×*g* for 2 h. Viral particles were resuspended in the LCP basal medium. Viral titers were measured using fluorescence expression in HEK293T cells (Barde et al., 2010). A series of dilutions of the virus were applied to HEK293T cells with 8 μg/mL polybrene (Sigma) and incubated for 72 h. The HEK293T cells were then analyzed using flow cytometry to quantify the percentage of cells expressing the fluorescent marker. Titer was calculated from the dilution that yielded between 1 and 20% of cells positive.

### Applications of LCPs

#### Drug Screening Using LCPs

To assess the effect of drug candidates on cell proliferation, the cells were fed with basal medium containing growth factors supplemented with CHIR and/or drug for 10 days. For differentiation analysis, the cells were maintained in proliferation medium for 10 days and then switched to basal medium containing LY, CHIR and/or drug for an additional 10 days. All the examined drugs were tested in triplicate. Samples were analyzed using the Accuri flow cytometer (BD Biosciences) and FlowJo (Accuri software) or, alternatively, harvested for gene expression analysis using quantitative real-time PCR (qRT-PCR). To generate additional wells for larger scale screening, each cochlea can be divided into four wells of a 96-well plate in 15 ul Matrigel droplets.

#### Lentiviral Transduction of LCP Cells

Cochleae were dissected and processed for LCP generation as described above. After straining through a 40-micron cell strainer, cells were counted using a hemocytometer. Cells were transduced by combining the single-cell suspension and concentrated lentivirus as needed to reach the desired titer, in LCP basal medium supplemented with 8 μg/mL polybrene (Sigma). Viruses used in this study include the GFP-expressing virus (FUGW was a gift from David Baltimore, Addgene #14883; Lois et al., 2002) and the sgRNA constructs described above. Transduction was performed under centrifugation at 600 ×*g* for 30 min in a centrifuge preheated to 33°C. After centrifugation, the supernatant was decanted, and the cells were resuspended in 100% Matrigel and mounted in 24-well plates as described above.

#### Manipulation of LCPs and Neuro2a Cells With CRISPR/Cas9

Neuro2A cells were maintained with DMEM supplemented with 10% FBS (Biological Industries). To validate the specificity of the gRNA prior to LCP transduction, Neuro2A cells were co-transfected with a *Cas9* expression plasmid pLV-hUbC – Cas9-T2A-GFP (Addgene, #53190) and gRNA expression lentiviral vector using Lipofectamine 2000 (Invitrogen) according to the manufacturer’s protocol.

In LCPs, expression of *Cas9* in combination with cell tracing was achieved by crossing *Sox2-CreER* mice to both *CAG-flox-tdTomato* reporter mice and *CAG-flox-Cas9* mice. Administration of tamoxifen induced Cre recombinase-mediated excision of the floxed regions, allowing for the expression of *Cas9* and the red fluorescence gene *tdTomato* as a reporter. LCPs were generated from the triple transgenic mouse and transduced with the gRNA lentivirus targeting *Notch1* as described above.

### T7 Endonuclease Assay

After manipulation using CRISPR/Cas9, LCPs and Neuro2A cells were incubated for 5 days, and genomic DNA was isolated using the DNeasy Blood and Tissue Kit (Qiagen) following the manufacturer’s instructions. 50 ng of genomic DNA was used as the template in PCR reactions to amplify the targeted genomic loci with the flanking survey primers: GTGTGCGTCAACGTCCGAT (forward) and CAACGAGAGTATAGCGCCCC (reverse), using Q5 High-Fidelity DNA Polymerase (NEB). PCR products were purified with a Monarch DNA Gel Extraction Kit (NEB). 250 ng of purified PCR product was combined with NEBuffer 2 (NEB), denatured and then re-annealed in a thermocycler by cooling from 95 to 25°C at a rate of 0.1°C/s. The re-annealed DNA was incubated with 1 μl of T7 Endonuclease I (10 U/μl, NEB) at 37°C for 15 min. The samples were then analyzed on a 5% TBE 18-well Criterion PAGE gel (Bio-Rad) electrophoresed for 30 min at 200 V and stained with EtBr for 20 min. Cas9-induced cleavage bands and the uncleaved band were visualized on a ChemiDoc Imaging System (Bio-Rad).

### Analysis of Expanded and Differentiated LCPs

#### RNA Extraction and Quantitative Real-Time PCR (qPCR)

For expression analysis using qRT-PCR, LCPs were incubated with Matrisperse Cell Recovery Solution (Corning) for 1 h at 37°C in order to disrupt the Matrigel. Solution was then removed, and the cells were resuspended and frozen in RLT lysis buffer (Qiagen). RNA was extracted using RNeasy Micro Kit (Qiagen) and cDNA was prepared using ImProm-II Reverse Transcription kit (Promega) according to the manufacturer’s instructions. qPCR was performed in triplicates using FastStart Universal Probe Master Mix (Roche) and the following TaqMan assays (Applied Biosystems): Atoh1 Mm00476035_s1; Myo7a Mm01274015_m1; Lgr5 Mm00438890_m1; Ccnd1 Mm00432359_m1. Gapdh Mm99999915_g1, was used as internal control.

#### Flow-Cytometry

Flow cytometry was used to evaluate the number of GFP-positive LCPs at different stages of expansion and differentiation. *Lgr5-EGFP-IRES-CreER* mice were used to label Lgr5-positive cells for analysis during proliferation, while *Atoh1-nGFP* mice were used to label LCPs for analysis during differentiation. To generate single-cell suspensions, LCPs were incubated 1 h with Matrisperse Cell Recovery Solution (Corning) followed by 20 min with TrypleE, both at 37°C. After removal of the TrypLE, the cells were resuspended and triturated in HBSS containing 2% FBS and analyzed using FACSAria II (BD Biosciences) or Accuri flow cytometer (BD Biosciences) and FlowJo (Accuri software).

### Statistical Analysis

All experiments were repeated 3–5 times as reported. Results are presented as average percentage of positive cells or average fold change vs. control with standard error of the mean. Statistical significance was established using Student’s *t*-test or one-way ANOVA depending on the number of variables tested and *p*-value ≤ 0.05 was considered significant. Statistical analysis was performed using GraphPad PRISM 7.

## Results

### Differentiated LCPs Upregulate *Atoh1* and *Myo7a*, Similar to Native Hair Cells

In the developing cochlea, *Atoh1* expression begins at E13.5 and the first hair cells can be detected at E14.5 at which time *Myo7a* expression is first detected (Pan et al., 2012). *Atoh1* peaks around birth and later decreases as the hair cells mature, while *Myo7a* increases throughout development and is a characteristic marker of adult hair cells. To evaluate whether LCPs could be used to model hair cells while mimicking the same differentiation pattern *in vitro*, LCPs were generated from *Atoh1-nGFP* reporter mice, expanded for 10 days (D0) and differentiated for 1–10 days (D1–D10). Expression levels were analyzed in triplicate from litter-matched and culture-matched samples. *Atoh1* was significantly upregulated as early as 1 day after initiation of differentiation. Its expression increased until D3-D4 and then decreased over the following few days (D5–D10) of differentiation (Figure 1B). In contrast, *Myo7a* increased more gradually and continuously over 10 days of differentiation (Figure 1C). *Atoh1* and *Myo7a* expression patterns suggest a similar path driving both LCPs and native hair cell differentiation. In support of a shift from proliferation to differentiation, expression analysis of *Lgr5* confirmed previous findings (McLean et al., 2017), in which *Lgr5* expression is reduced, indicating that the Lgr5-positive cells are differentiating to hair cells (Figure 1D). Additionally, analysis of the cell cycle gene and supporting cell marker *Ccnd1* (Laine et al., 2010) indicated a decrease consistent with cell-cycle arrest (Figure 1E). It should be mentioned that native *Atoh1* expression using immunohistochemistry could not be evaluated at the required sensitivity and while McLean et al. demonstrated Myo7a expression at D10 (McLean et al., 2017), we focused here on more subtle changes at earlier timepoints during differentiation.

### LCPs as a Platform for Drug Screening

We assessed the effects of some drug candidates on the proliferation or differentiation of LCPs. We adapted the LCP protocol to conduct a screen of several compounds, known to influence specific cell signaling pathways or epigenetic dynamics. The drug effects were analyzed by flow cytometry and verified by qPCR. Both supporting cell proliferation and differentiation are necessary for the goal of regenerating hair cells, and drugs were tested for either or both effects in the *in vitro* progenitor cells. This protocol yielded an average of ∼30,000 Lgr5-positive cells per well at the end of the expansion stage and ∼12,000 Atoh1-positive cells per well at the end of the differentiation stage.

**FIGURE 2:**
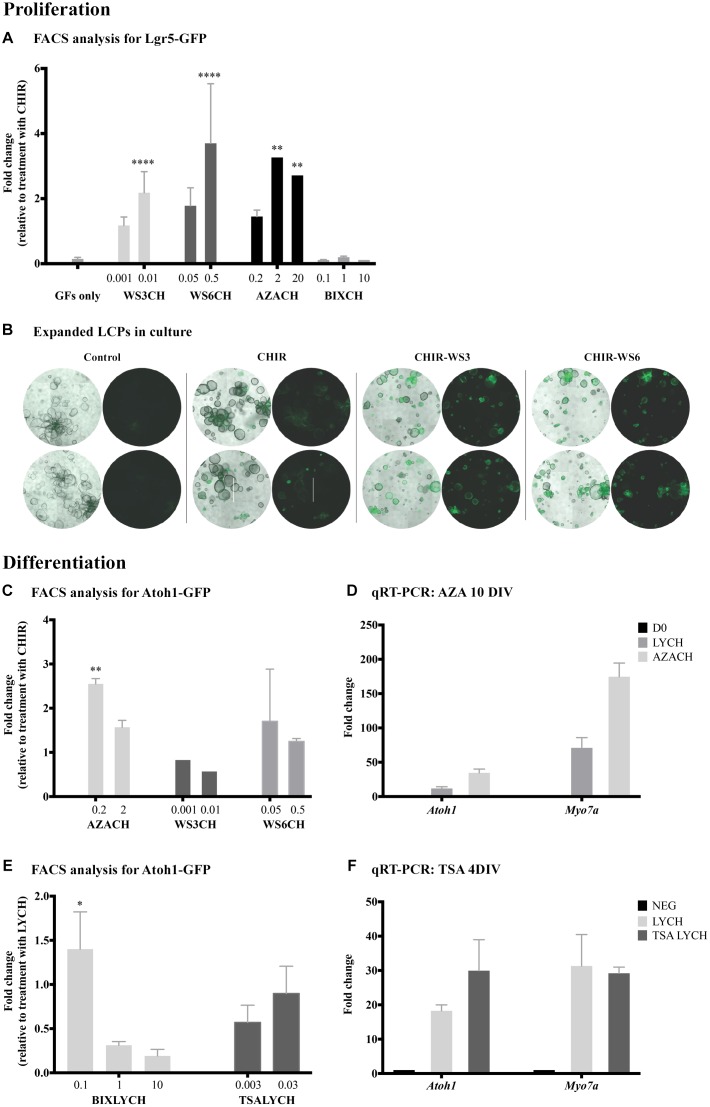
Identification of new drugs that induce proliferation and differentiation. **(A)** Flow cytometry analysis of Lgr5-positive cells after 10 days of proliferation. Addition of WS3, WS6 and 5-azacytidine (AZA) had no affect alone, but significantly increased proliferation yield when combined with CHIR, compared to CHIR alone. **(B)** Bright-field images of expanded LCPs in basal medium and in combination with CHIR and WS3 and WS6. In the presence of CHIR alone, the organoids appeared larger compared to control basal medium and a portion expressed Lgr5. While the organoids appeared smaller when WS3 or WS6 were added to CHIR, the number of Lgr5-positive colonies increased substantially. Scale bar: 500 μm. **(C,D)** Treatment with 5-azacytidine in combination with CHIR resulted in the highest increase in differentiation compared to CHIR alone, as evaluated using flow-cytometry analysis of Atoh1-positive cells **(C)** and validated using qPCR expression analysis of *Atoh1* and *Myo7a* **(D)**. **(E)** Treatment with BIX01294 in combination with LY-CHIR resulted in the highest increase in differentiation compared to LY-CHIR, while treatment with trichostatin A (TSA) in combination with LY-CHIR resulted in a similar degree of differentiation as LY-CHIR. **(F)** Expression analysis of *Atoh1* and *Myo7a* using qPCR demonstrated increased expression of *Atoh1* and decreased *Myo7a* after treatment with TSA. Results are presented as average fold change ± SEM; ^∗^*p*-value ≤ 0.05; ^∗∗^*p*-value ≤ 0.01; ^∗∗∗∗^*p*-value ≤ 0.005 calculated using one-way ANOVA. All drug concentrations are in μM. Growth factors (GF); CHIR99021 (CH). Addition of CH at the end of a drug name or initial indicates the treatment combination of the drug and CHIR99021.

In tests for proliferation of LCPs, flow cytometry was used to quantify the relative number of Lgr5-GFP-positive cells under different drug conditions. Since the expansion medium includes several compounds, potentially interfering with the effects of a candidate drug, the control for expansion was CHIR alone (defined as 1). Treatment with the methyltransferase inhibitor 5-azacytidine (A2385, Sigma-Aldrich) in combination with CHIR resulted in a threefold increase in Lgr5-positive cells at both 2 and 20 μM (Figure 2A). Additionally, the inhibitors of the ErbB3-binding protein, WS3 and WS6 (SML0758 and SML0757, respectively; Sigma-Aldrich), in combination with CHIR, had a pronounced concentration dependent effect on LCP proliferation. Concomitantly, bright-field observation of organoid shape and Lgr5-GFP fluorescence after treatment with WS3 and WS6, indicated an increase in Lgr5-positive colonies compared to CHIR and growth factors alone (Figure 2B). In contrast, treatment with the G9a inhibitor BIX01294 (B9311, Sigma-Aldrich) in combination with CHIR, resulted in inhibition of proliferation as demonstrated by a lower percentage of Lgr5-positive cells.

To assay for differentiation of LCPs toward a hair cell fate, we derived the cells from *Atoh1-nGFP* mice and quantified Atoh1-GFP-positive cells under different conditions. Our basal differentiation medium included the Notch-pathway inhibitor LY and the Wnt-pathway activator CHIR (McLean et al., 2017). We therefore sought to understand the effect of candidate drugs on differentiation alone, or in combination with CHIR and/or LY. In contrast to the proliferation stage, CHIR supplemented with WS3 or WS6 showed no further effect than CHIR alone, suggesting that ErbB signaling influenced proliferation rather than differentiation of LCPs (Figure 2C). In contrast, treatment with 5-azacytidine in combination with CHIR resulted in a 2.5-fold increase in Atoh1-GFP-positive cells compared to CHIR alone. Analysis for *Atoh1* and *Myo7a* in 5-azacytidine treated samples supported the flow cytometry finding of increased expression of the 2 markers after both treatments (Figure 2D). None of the tested drugs significantly increased differentiation on their ’own.

BIX01294 addition to LY-CHIR resulted in a highly significant increase in differentiation (Figure 2E). The maximum effect was seen at a low concentration, suggesting either a toxic or contradictory effect in LCPs at high concentrations. Addition of VPA during LCP differentiation did not increase the hair-cell yield (data not shown). Treatment of expanded LCPs with LY-CHIR supplemented with another HDAC inhibitor, trichostatin A (TSA, T8552, Sigma-Aldrich), similarly had no effect on differentiation, but resulted in a significant decrease in Atoh1-positive cells at low concentrations. However, TSA enhanced expression of the hair cell differentiation marker *Atoh1* compared to the standard LY-CHIR differentiation medium, 4 days after onset of differentiation (Figure 2F).

### LCPs Can Be Transduced in High-Yield

**FIGURE 3:**
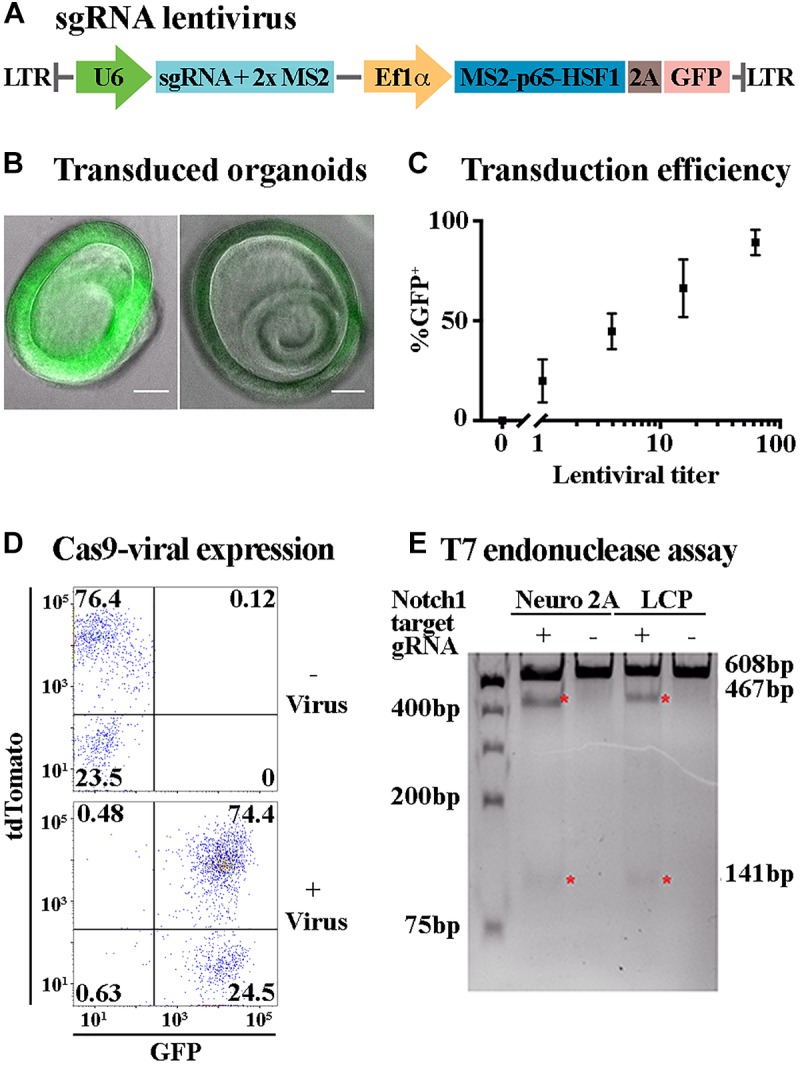
Lentiviral transduction enables ectopic expression and genome editing in LCPs. **(A)** Schematic representation of the plasmid used for sgRNA viral transduction. hU6, human U6 promoter. hUbC, human ubiquitin C promoter. **(B)** Representative bright field images of expanded LCPs after transduction with lentivirus. Intensity of the fluorescence from the transgene (green) varied between experiments. Scale bar: 100 μm. **(C)** LCPs transduction efficiency varied in proportion to the titer of the lentivirus that was delivered to the cells. Relative number of recovered LCPs after transduction was measured using the expression of GFP from the transgene and correlated to the titer delivered per cell. The titer was measured based on the infection of HEK293T cells prior to LCP transduction. Results are presented as mean ± SEM. **(D)** Flow cytometry analysis demonstrated overlapping expression of tdTomato and sgRNA-GFP virus after Sox2-driven Cre activation in expanded LCPs. tdTomato was measured as a marker for Cas9 expression after Cre activation with tamoxifen, with similar percentage of positive cells in the presence and absence of the virus. Extensive viral transduction is evident by the 98.9% of cells that were GFP positive. 74.4% of the cells were positive for both tdTomato and GFP, indicating high yield of transduction into Cas9 expressing LCPs, enabling subsequent activity of Cas9 in conjunction with the transduced sgRNA. **(E)** T7 endonuclease assay was used to validate Cas9/gRNA activation at the target site of *Notch1* in Neuro2A and LCPs. While *Cas9* is expressed in all cells after Cre activation, two distinct cut bands were detected only in the presence of the *Notch1* gRNA denoting the specificity of the gRNA in this locus (red asterisk).

Lgr5-positive cochlear progenitors can be generated from any mouse model thus enabling genetic modifications of gene expression, including gene knock-out and over-expression with spatial and temporal control using the Cre/loxP and tet systems (Nagy, 2000; Bockamp et al., 2002). Nevertheless, the number of mouse models is limited and their generation is costly. Alterations in gene expression patterns and combination of multiple perturbations can be more effectively achieved using viral transduction. Viral transduction requires prolonged incubation of the target cells with the viral particles. Incubation of Matrigel embedded LCPs did not result in a sufficiently high yield (data not shown). Therefore, an additional step was added to the LCP generation, whereby single-cell suspension was combined with concentrated lentivirus and centrifuged prior to embedding in Matrigel. Substantial transduction was achieved, using a GFP fragment packaged in lentivirus, reaching as many as 98.9% GFP-positive cells, as measured by flow-cytometry (Figure 3D). The expanded organoids were distinctly labeled, though in varying intensities (Figure 3B). Titer examination indicated a linear correlation between the lentiviral titer and the percentage of GFP-positive cells, with 100 viral particles per cell resulting in approximately 100% transduction efficiency (Figure 3C).

### LCPs as a Platform for CRISPR/Cas9 Analysis

The ability to transduce LCPs in high yield enabled the use of CRISPR/Cas9 methodologies to further manipulate gene expression during expansion and differentiation. For an initial proof of concept, mice carrying *CAG-flox-Cas9* crossed with *CAG-flox-tdTomato* and *Sox2-CreER* were used for LCP generation. Since *Sox2* is expressed in Lgr5-positive expanded LCPs (McLean et al., 2017), treatment with 4-OH-tamoxifen resulted in Cre activation and subsequent presumed simultaneous expression of *tdTomato* and *Cas9*. *Cas9* expression in the cells was therefore evaluated through the expression of *tdTomato* that was detected in more than 76% of the cells (Figure 3D). For silencing using CRISPR/Cas9, LCPs were transduced with a virus containing sgRNA-GFP (Figure 3A). Viral transduction into *Sox2-CreER; CAG-flox-Cas9; CAG-flox-TdTomato* mice resulted in over 74% tdTomato-GFP-positive cells, indicative of *Cas9* and sgRNA combined expression that is required for their function (Figure 3D). T7 endonuclease activity assay was used to evaluate the functionality of the Cas9-sgRNA complex. The specificity of the sgRNA was first determined in Neuro2A cells using combined transduction of a lentivirus that carried *Cas9* and another lentivirus that carried a guide RNA for *Notch1*. Indeed, transduction of the *Cas9* alone did not result in any activity, as apparent by the single uncut *Notch1* band at 608 bp. However, upon the transduction of both *Cas9* and sgRNA-*Notch1*, 2 additional cut bands were detected at 467 and 141 bp, indicative of a Cas9-sgRNA mediated cleavage (Figure 3E). This experiment was then repeated using LCPs that were generated from *Sox2-CreER; CAG-flox-Cas9; CAG-flox-TdTomato* mice. After Cre activation, the LCPs were transduced with the sgRNA-*Notch1* virus. A similar outcome was detected, corroborating the feasibility of CRISPR/Cas9 mediated silencing in LCPs.

## Discussion

We have previously demonstrated LCP differentiation into hair cells in high yield and confirmed the expression of key hair cell markers, including the outer hair cell marker *Slc26a5* (prestin*)*, the inner hair cell marker *Slc17a8* (vGLUT3) and the transduction channel *Tmc1*, suggesting partial maturation of these new hair cells. Here we present additional evidence, whereby the *Atoh1* and *Myo7a* upregulation pattern mimics their induction during development, concurrent with *Lgr5* and *Ccnd1* downregulation. The increase in *Atoh1* transcript, followed by *Myo7a*, occurs during the first 4 days of LCP differentiation, analogous to their expression between E12.5 and P0 of inner ear development. The subsequent decrease in *Atoh1* and continued elevation of *Myo7a* are comparable to early postnatal stages (Pan et al., 2012). Comprehensive evaluation of hair cell maturation by electrophysiological measurements requires access to the apical surface of the cell, which is within the organoid. Due to the significantly smaller size of our organoids, these measurements will require development of a different approach than that used for organoids derived from embryonic stem cells (Koehler et al., 2017).

We also present an elaboration of our existing protocol that permits evaluation of drug candidates for expansion and differentiation of Lgr5-positive supporting cells. New methods to induce differentiation are important because the hair cells of the mammalian inner ear lack regenerative capacity. Specifically, testing drugs that promote proliferation during LCP expansion may serve as a platform for inducing supporting cell proliferation *in vivo*. Indeed, regulators of the receptor tyrosine kinase, ErbB, WS3 and WS6, in the presence of Wnt pathway activator, CHIR, produce a high yield of Lgr5-positive cells. These drugs inhibit the ErbB3-binding protein (EBP1), thus enhancing proliferation of pancreatic (Shen et al., 2013) and retinal cells (Swoboda et al., 2013). In the murine inner ear, expression of ErbB-associated genes in supporting cells has been linked to extended proliferative capacity and EGFR-dependent regeneration (White et al., 2012), particularly in greater epithelial ridge and pillar cells, where expression of both *Lgr5* and ErbB-associate genes have been observed (Shi et al., 2012). An interplay of Wnt signaling and ErbB signaling has been suggested to occur both during development and in cancer (Hu and Li, 2010), as seen in LCPs with a combined treatment of CHIR and WS3/6. Collectively, these findings suggest potential pathways that may induce supporting cell proliferation in the mammalian cochlea.

The epigenetic signature of a cell is influenced by DNA methylation and histone modifications. In an effort to identify epigenetic targets for hair cell regeneration, we screened a collection of epigenetic modifiers. Comprehensive analysis of each drug was performed in the presence and absence of key components of the medium. Histone modifiers (HDAC-TSA and G9a-BIX01294) and DNA methyltransferase (DNMT-5-azacitidine) inhibitors showed activity in supporting cell proliferation and hair cell differentiation. BIX01294 reduces the levels of the repressive mark H3K9me2 (Kubicek et al., 2007), while TSA increases the levels of the activating mark H3K9ac. Our findings revealed that inhibition of G9a using BIX01294 significantly inhibited proliferation but promoted differentiation at low concentrations. A recent study revealed that BIX01294 reduced supporting cell proliferation and hair cell regeneration in zebrafish, due to a decrease in H3K9me2 (Tang et al., 2016). While the discrepant findings could be attributed to the model (mouse vs. zebrafish), it is evident that different concentrations of BIX01294 may lead to opposing results. Thus, the effects of BIX01294 exposure and subsequent changes in H3K9me2 levels on hair cell differentiation warrant further study.

In LCPs, the HDAC inhibitor VPA in combination with CHIR, induces progenitor expansion as well as proliferation of supporting cells and differentiation of new hair cells in explants (McLean et al., 2017). This induction of proliferation could be related to the contribution of VPA to Notch1 activation (Wang et al., 2015). Short (24 h) incubation of postnatal cochlear explants with other HDAC inhibitors increased *Atoh1* expression in Atoh1-positive sorted cells (Stojanova et al., 2015). We observed a similar phenomenon, whereby TSA treatment increased *Atoh1* mRNA levels, but not the number of Atoh1-positive cells. *Atoh1* function in early hair cell development and its downregulation during hair cell maturation, accompanied by the role of VPA in proliferation, may imply that the increase in *Atoh1* in P1 explants as well as in LCPs at D4 results from maturation delay, induced by HDAC inhibitors such as VPA or TSA.

DNA methyltransferases (DNMTs) catalyze the transfer of additional methyl groups from S-adenosyl methionine (SAM) to 5′-cytosine in DNA, to silence gene expression. Promoter methylation by DNMTs interferes with the binding of transcription factors, repressing gene transcription. 5-aza-2′-deoxycytidine (5-Aza) is a potent inhibitor of DNMTs, incorporating into the genome and preventing DNA methylation in newly divided cells. While the effects of 5-Aza are genome-wide, its activity at promoter regions can reverse gene silencing. A significant effect on LCPs was observed upon treatment with 5-Aza combined with CHIR, with maximum effects on proliferation and differentiation occurring at different concentrations. Interestingly, addition of 5-Aza while the LCPs were actively dividing (day 7 of proliferation) had the greatest effect on differentiation. These findings support previous findings of enhanced expression of epithelial genes with DNMT inhibition (Zhou and Hu, 2015).

In addition to small molecule-based manipulations, we have developed a protocol for gene delivery to LCPs. Our protocol allows us to transduce upward of 95% of our harvested cell culture using only laboratory-scale lentivirus production. While *in vivo* CRISPR/Cas9 experiments may require transitioning to other viral types (Gao et al., 2018), for *in vitro* analysis, lentivirus has a sufficient packaging capacity to carry large cargo including the *Streptococcus pyogenes* Cas9 protein, enabling precise, long-term and economical genomic perturbations, as described here. Our protocol relies on the use of third-generation lentivirus, which is packaged in the absence of the HIV *tat* protein, thus avoiding interactions between *tat* and the Notch pathway, evident in the use of second-generation packaging plasmids (Shoham et al., 2003; Fan et al., 2016).

A limitation to any macromolecular delivery into LCP culture is the physical barrier presented by Matrigel. Viral particles and liposomes have a limited ability to diffuse into the 3D culture, necessitating the development of a protocol that could deliver macromolecules prior to embedding in Matrigel. This issue was successfully resolved by delivering virus to LCPs at the start of the expansion phase; however, investigations requiring delayed or otherwise precise temporal control of gene expression will necessitate the inclusion of inducible genetic elements such as the estrogen receptor element or tet systems.

We demonstrated here the use of LCPs as a model for *in vitro* manipulation of hair cell differentiation and a tool to study both supporting cell proliferation and hair cell generation using genetic and pharmacological alterations.

## Author Contributions

All authors designed and performed the research, analyzed the data, wrote the manuscript, and have read and confirmed this publication.

## Conflict of Interest Statement

AE is a co-founder and consultant to Decibel Therapeutics. The remaining authors declare that the research was conducted in the absence of any commercial or financial relationships that could be construed as a potential conflict of interest.

## Footnotes

1 http://www.who.int, 15 March 2018.
